# Questionnaire-Related Deferrals in Regular Blood Donors in Norway

**DOI:** 10.1155/2012/813231

**Published:** 2012-01-17

**Authors:** Håkon Reikvam, Kjersti Svendheim, Anne S. Røsvik, Tor Hervig

**Affiliations:** ^1^Department of Haematology, Institute of Internal Medicine, University of Bergen, 5021 Bergen, Norway; ^2^Department of Immunology and Transfusion Medicine, Haukeland University Hospital, 5021 Bergen, Norway; ^3^Gades Institute, University of Bergen, 5021 Bergen, Norway

## Abstract

Voluntary donation is a key issue in transfusion medicine. To ensure the safety of blood transfusions, careful donor selection is important. Although new approaches to blood safety have dramatically reduced the risks for infectious contamination of blood components, the quality and the availability of blood components depend on the willingness to donate and the reliability of the information given by the donors about their own health, including risk behavior. As donors who are deferred by the blood bank will be less motivated to return for donation, it is important to reduce the number of deferrals. The aims of the present study were to investigate the reasons for deferral of registered donors coming to the blood bank for donation, in order to identify areas of importance for donor education—as these deferrals potentially could be avoided by better donor comprehension. Deferral related to testing of donors is not included in this study as these deferrals are dependent on laboratory results and cannot be indentified by questionnaire or interview. Data were collected from all blood donors in a period for 18 months who came for blood donation at a large university hospital in Norway. 1 163 of the 29 787 regular donors, who showed up for donation, were deferred (3.9%). The main reasons were intercurrent illness (*n* = 182) (15.6%), skin ulcers (*n* = 170) (14.6%), and risk behaviour (*n* = 127) (10.9%). In a community, intercurrent illnesses, skin ulcers, and potential risk behavior are the most frequent reasons for deferral of regular donors. Strategized effort on donor education is needed, as “failure to donate” reduces donor motivation.

## 1. Introduction

The blood donor is the key to patient safety. The “ideal blood donor” is a voluntary, nonnumerated, repeat donor—as this donor will have the lowest risk of having an infection that may be transmitted through blood transfusion. Thus, a major task for the blood banks is to take care of the regular donors. An important part of this strategy is to avoid donor deferrals, as donor deferral imposes a risk of “no-return” of the donor. The more the donor knows about reasons for deferral, the better will the chance be that the donor will not appear in the donation room in a state related to temporary deferral. Thus, donor education is essential, as is also stated in the European Blood Directive [1]. Already in the early days of the HIV/AIDS epidemics, donor knowledge on risk factors was known to be important to avoid transmission of infection [2]. As the donor questionnaire has been more and more complicated, the donors need to have a broad knowledge on transfusion-related risks to avoid “dry visits” to the blood bank. This is also underlined by the extensive requirements for donor education as listed in the European Blood directive [1]. In Norway, the need to reduce donor deferral is especially important, as the country has a donor shortage even though the blood utilization is at average European level. During the last years, the yearly donation frequency has been between 2.4 and 2.2, far from the goal of 1.5–1.7 donations per year. Despite the need to minimize donor deferral, no national data enable us to reduce this negative factor for blood donation is available in Norway.

Thus, the purpose of this study was to provide useful information to improve the communication with the donors—both in contact with the registered donors and in “donor education sessions” for new donors [3].

## 2. Material and Methods

Data were collected from all donors who appeared at the Blood Bank, Haukeland University Hospital between June 15, 2008 and December 15, 2009. This is the largest Blood Bank in Western Norway, with approximately 23,000 donations, including platelet and red cell apheresis. As for all 41 Norwegian blood banks (total number of donations 220,000), all donors are repeat donors and only registration procedures are performed at the first visit to the blood bank.

At all 29,787 blood donations, a written questionnaire about on health condition and potential risk behaviour was filled in. The data presented in this study simply reflect the answers the donor filled in, with supplementary information obtained during the blood donor interview. The data collection was performed by going through all the questionnaires for the actual period. Persons who were deferred from donation because of low haemoglobin concentration, low ferritin concentration or blood pressure, and pulse outside the approved limits were not included in the study, as we consider these parameters to be beyond the topics that may be picked up by the questionnaire. Retrospectively all reasons for donor deferral were evaluated and classified. Descriptive statistics were performed by the use of SPSS (SPSS Inc., Chicago, IL, USA). Graphical presentations were made by using GraphPad Prism 4 (Graph Pad Software, Inc., San Diego, CA, USA).

## 3. Results

**Figure 1:**
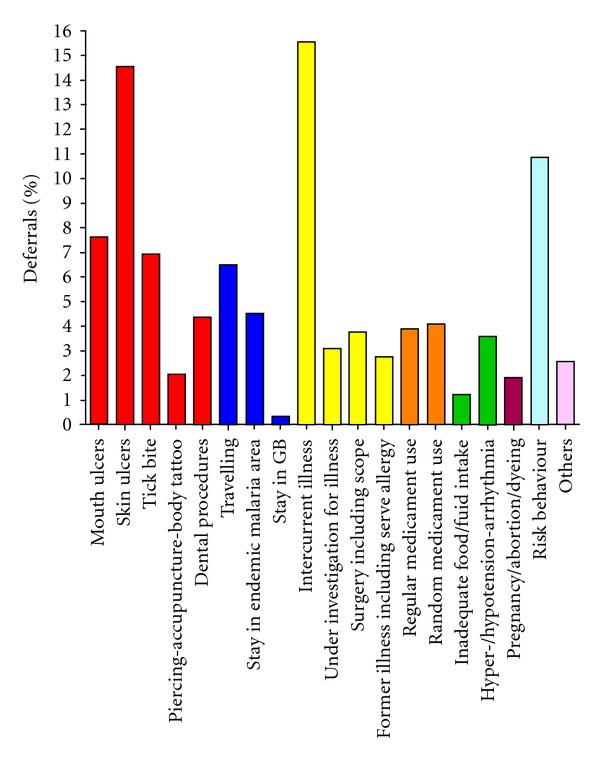
Reasons for refusing 1163 potential blood donors for donating. The figure demonstrates the classification of patients deferred from blood donation given as percentage of total deferrals. Red colour indicates deferrals reasons related to affection of skin barrier, blue indicates reasons related to abroad stay, yellow indicates disease-related deferrals, orange deferrals related to use of pharmacological agents, green physical reasons for deferral and pregnancy and related deferrals, and risk behaviour and others are indicated with own colours.

**Figure 2:**
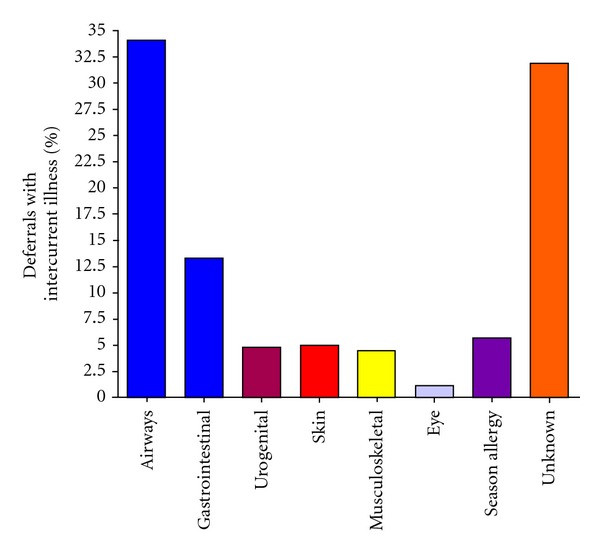
Subdividing of potential donors rejected because of intercurrent illness. The figure demonstrates the affected organ system or aetiology of the refused donors of intercurrent illness.

**Figure 3:**
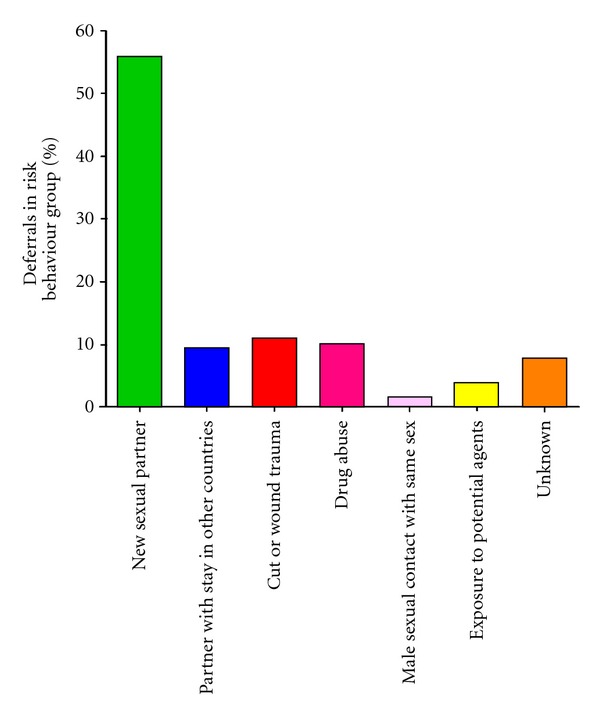
Subdividing of potential donors rejected because of risk behaviour. The figure demonstrates the reasons of the refusing donors with classified risk behaviour.

1163 donors (repeat donors) were deferred from donation. The total deferral rate was thus 3.9%. There were multiple reasons for donor rejection, as summarized in Figure 1. The main reason is *intercurrent illness* (*n* = 182) (15.6%), skin ulcers (*n* = 170) (14.6%), and risk behaviour (*n* = 127) (10.9%). Because intercurrent illnesses and risk behaviour are two of the largest groups among the refused donors, these two groups were further analyzed and subdivided. The underlying illnesses were classified according to aetiology or organ system affected (Figure 2). The most common reasons were respiratory tract infections (*n* = 62) (34.1%), gastrointestinal symptoms (*n* = 24) (13.2%), and exaggeration of season allergy (*n* = 10) (5.5%), in addition to a large group of unknown or unclassified reasons (*n* = 58) (31.9%). The group classified as “risk behaviour” was further analyzed (Figure 3). The dominant cause in this group was change of sexual partner (*n* = 71) (55.9%). Only 160 donors (0.6% of the total donations, 1.6% of the total donor base) were permanently excluded due to medical reasons during the registration period.

## 4. Discussion

The background for the blood donor criteria is to ensure that the donation does not pose any risk to the donor and to prevent the patient receiving blood components to be exposed to risk related to the donor [3]. Thus blood donor education is important as knowledge may prevent unsuitable individuals from becoming blood donors and the registered donors from coming to the blood bank when they may not be allowed to donate due to a temporary deferral reason [3]. Major reasons for temporary deferral are low haemoglobin concentration and/or depleted iron stores in the donor. As these factors are not directly related to donor education, these subjects are not discussed in this paper. We have implemented an “iron conservation program”, which is referred to in other papers [4, 5]. The “donor education” principle is not new; on the contrary during all times of blood donation there have been a lot of pamphlets produced and verbal information has been given to the donors on many occasions [6]. The new aspect is the formal approach to blood donor education, as being highlighted in the European Blood directive [1]. Donor education is, however, no guarantee for better donor [7].

On the other hand, donor education must be precise to achieve reduced risks for the patients [8]. All in all, the total rejection rate of 3.9% as reported in this paper may seemingly be acceptable and in line with data from earlier reports [9]. The main reasons for rejections were intercurrent disease, mostly related to upper airway infection—and wound infection. The term “intercurrent disease” refers to several not severe diagnoses, as shown in Figure 2. Of these diagnoses, gastrointestinal disease may be the nearly only of importance to the patient. It is well known that infection by Yersinia enterocolitica may cause only minor gastrointestinal symptoms in a person even if bacteraemia is present [10]. Accordingly, there are several reports of Yersina-infected donors leading to disease—and sometimes death—in the related blood recipient [11]. Therefore, several blood transfusion services introduced 2-3 weeks quarantine for donors with a history of diarrhoea. However, as this symptom is not well defined, and the frequency of “bad stomach” is high, this precaution has been abandoned by some blood services. “Skin problems” is another frequent cause of deferral of regular blood donors. There is established knowledge that wounds may be “subclinical infected”—and that this infection is followed by undiagnosed bacteraemia. The probability that a wound in a donor is related to bacteraemia is however minor [12].

Also, the importance of other causes of low-grade bacteraemia to blood transfusion is difficult to access. For Borrelia infections, which are common in Norway, no proof of blood transfusion-related transmission is available [13]. Even minor dental procedures may lead to temporary bacteraemia [14], but it would be impossible to implement quarantine periods for donors brushing their teeth.

Figure 3 shows the risk factors indentified—meaning the conditions where the donor has increased risk of carrying an infection that may be transferred through transfusion. The most frequent condition on this list is “change of sexual partner”. In Norway, a blood donor who changes sexual partner is deferred for six months. If the new sexual partner does not belong to any risk group as listed in the donor questionnaire, the real impact on donor safety by this subject is disputable indeed. In Norway, it has earlier been reported that the blood donors have “low-risk behaviour” [15], thus contrasting a more recent report from Brazil [16]. It has also been indicated that the combination of a written questionnaire and an oral face-to-face interview does not ensure that the donors provide all relevant information [17]. In some countries, temporary deferral for change of sexual partner is for a very short period only or not implemented at all. Seemingly, there is need for more consensuses concerning new sexual partner as reason for donor deferral.

Other factors identified are related to “low risk”. Again, in parallel with intercurrent skin disease, skin infection comes on the list. Sound identification of “wounds at risk” that is subclinical wound infections will probably be impossible. The best option may be to inform donors not to show up at the blood bank if the donor actually has a wound, although this may cause problems for donors working, for example, as carpenters. Thorough education of blood bank staff may also be of help, although it is difficult also for health professionals to identify “dangerous” wounds. Encouraging the donors to call back if infectious symptoms occur later may also be useful, also because this is a general principle not just only related to wound infections.

It is interesting that a few male donors report sex with other men. Even though disputed by some blood transfusion services, this is in general accepted as a real risk factor [18], and it is well communicated that males with male sexual contact should not donate blood. It is not known why this risk factor is sometimes overlooked by the donors—as the donors sometime forget recently holidays and appear in the blood bank shortly after being exposed to malarial infection—and do not inform the blood bank about it.

A limitation of the study is due to the practical approach we have used, and the study is therefore too small to provide data on factors as age, gender, and ethnicity in relation to donor deferral. For information concerning these matters we refer to a publication by Custer et al. [19].

In conclusion this survey of deferral reasons identifies that most rejections are not related to conditions which put the patient receiving blood components at high risk, but better donor knowledge on reasons for temporarily deferral could reduce the “dry visit” frequency, which again could improve donor motivation [20]. The reported results will be presented for our regular donors and a new survey after two years to see if there is reduction in donor deferral. Thus, we hope to avoid the negative effects of short-term deferral on future blood donations [21].

##  Conflicts of Interests

The authors report that they have no potential conflicts of interest.
